# Gaze perception in social anxiety and social anxiety disorder

**DOI:** 10.3389/fnhum.2013.00872

**Published:** 2013-12-16

**Authors:** Lars Schulze, Babette Renneberg, Janek S. Lobmaier

**Affiliations:** ^1^Department of Educational Sciences and Psychology, Freie Universität BerlinBerlin, Germany; ^2^Institute of Psychology, University of BernBern, Switzerland; ^3^Center for Cognition, Learning and Memory, University of BernBern, Switzerland

**Keywords:** avoidance, cone of gaze, emotion, eye-tracking, eye gaze, mutual gaze, social anxiety, social anxiety disorder

## Abstract

Clinical observations suggest abnormal gaze perception to be an important indicator of social anxiety disorder (SAD). Experimental research has yet paid relatively little attention to the study of gaze perception in SAD. In this article we first discuss gaze perception in healthy human beings before reviewing self-referential and threat-related biases of gaze perception in clinical and non-clinical socially anxious samples. Relative to controls, socially anxious individuals exhibit an enhanced self-directed perception of gaze directions and demonstrate a pronounced fear of direct eye contact, though findings are less consistent regarding the avoidance of mutual gaze in SAD. Prospects for future research and clinical implications are discussed.

## Introduction

Social anxiety disorder (SAD) is one of the most common mental disorders with a lifetime prevalence of up to 12% in Western countries (Fehm et al., 2005; Kessler et al., 2005). Hallmark characteristics are intense fear and avoidance of being evaluated or criticized resulting in extreme discomfort and self-consciousness in everyday social situations (American Psychological Association, 2000). Theoretical models highlight the importance of cognitive biases in the processing of ambiguous or negative cues during social interactions for the etiology and/or maintenance of social anxiety (Rapee and Heimberg, 1997; Clark and Mcmanus, 2002). More specifically, studies show that socially anxious individuals have attentional biases in the processing of negative, rejection-related cues (Bar-Haim et al., 2007) and interpret ambiguous social situations as more threatening and negative than healthy controls (e.g., Stopa and Clark, 2000; Beard and Amir, 2009).

Relatively little attention, however, has been paid to biases in gaze perception. This is particularly surprising since individuals with SAD experience intense feelings of being looked at by other individuals and show a marked avoidance and fear of eye contact during social interactions (Schneier et al., 2011b). Biases in the self-referential perception of gaze directions, for instance, might more easily elicit feelings of mutual gaze and being the center of attention, which then will activate fears of being scrutinized by others. Here, we review studies with clinical and non-clinical socially anxious samples on self-referential and threat-related biases in the perception of mutual gaze.

First, mutual gaze perception in healthy human beings will be discussed. Next, biases in the perception of other individuals’ gaze in social anxiety will be reviewed with a focus on: (a) whether mutual gaze is more readily perceived; and (b) whether mutual gaze is avoided and perceived as threatening.

## Gaze perception in healthy human beings

Most mammals generally interpret direct gaze as threatening or as a sign of dominance. Humans in contrast often associate mutual gaze with positive interest, such as love and attraction. A preference for direct gaze seems to be present at a very early age: Farroni et al. (2002) found that infants as young as 2 days old prefer to look at faces that gazed directly at them compared to faces with averted gaze. Yet, humans sometimes find eye contact uncomfortable, for example if a stranger keeps staring at them.

Different sources of information are taken into account when processing gaze direction. The most obvious cue lies in the eye itself. Kobayashi and Kohshima (1997, 2001) compared the eyes of a large number of primates and found that the morphology of the human eye is rather unique. Of all compared species human eyes have the highest width to height ratio and the highest index of exposed sclera size. The amount of visible sclera provides information about the orientation of the eyeball (Gibson and Pick, 1963; Cline, 1967; Anstis et al., 1969; Langton et al., 2000; Ando, 2002). Ando (2002) provided direct evidence that the iris/sclera ratio is an important cue for eye gaze perception. By darkening one side of the sclera of eyes directed straight ahead, he found a substantial shift of the perceived gaze direction towards the darkened side.

Another factor influencing gaze perception is the head direction of the looker. Langton (2000) (see also Wollaston, 1824) found that the orientation of another person’s head strongly influenced the perceived direction of the person’s gaze. Body posture is yet another cue that can provide information about where someone is attending (Perrett et al., 1992).

Studies that focus on the ability to distinguish between direct and averted eye gaze are relatively numerous. All these studies generally report that human observers are highly accurate at determining mutual eye gaze. In their classic study, Gibson and Pick (1963) asked observers to indicate whether a “looker” who was sitting opposite was making eye contact or looking at a peripheral target. The authors found that an angular deviation of the eye by only 2.8° was correctly detected as not making eye contact. Cline (1967) replicated and extended these findings and reported that an angular deviation of as little as 0.75° was readily detected by an observer. Such high accuracy rates in detecting mutual gaze are not undisputed, since a number of studies found relatively poor discrimination of gaze direction, especially when the distance between looker and observer was large (e.g., Vine, 1971). With decreasing security (i.e., when visual information was reduced through distance or noise) observers tended to assume mutual gaze. There thus seems to be a considerable range wherein a person feels being looked at. Gaze direction might hence be better described as a cone rather than a ray (as assumed by e.g., Gibson and Pick, 1963; Cline, 1967). Consequently, Gamer and Hecht (2007) introduced the cone of direct gaze (CoDG) as a concept to measure mutual gaze perception. The authors found an average width of the CoDG of between 4° and 9° of visual angle, depending on the distance between looker and observer.

Facial emotional expression is another cue taken into account when judging gaze directions. Lobmaier et al. (2008) presented participants with three-dimensional models that were either facing the observer, or were rotated 2°, 4°, 6°, 8°, and 10° to the left and right. In this study eye gaze and head direction were aligned with each other (i.e., the whole head was rotated keeping the eyes relative to the head direction constant). Participants were asked to judge whether the face was looking at them or not. The results revealed a remarkable positivity bias: happy faces were more likely perceived as looking at the observer than angry, fearful, or neutral faces. The authors interpreted this finding in favor of self-esteem preservation: perceiving other’s happiness as directed at oneself is socially rewarding (see also Lobmaier and Perrett, 2011). This interpretation is compatible with the assumption that human beings have a prior expectation that other people’s gaze is directed towards them (Mareschal et al., 2013).

Ewbank et al. (2009) employed the CoDG metaphor to further test the influence of emotional expression on perception of direct gaze. Using the method of constant stimuli (see also Mareschal et al., 2013) angry, fearful and neutral faces were presented in which the direction of eye gaze was manipulated. They found that the CoDG was significantly wider for angry faces compared to neutral and fearful faces.

The studies reviewed above reveal that gaze perception plays an important role in social interactions and is modulated by several factors, such as head direction, interpersonal distance, or emotional facial expressions (see also reviews by Graham and LaBar, 2012; Carlin and Calder, 2013; for behavioral and neuroscientific findings of gaze processing and gaze-emotion interactions). Given that social interactions are affected in SAD, it is conceivable that social anxiety might be associated with impeded gaze perception. In the following sections we discuss gaze perception in the context of SAD.

## Gaze perception in social anxiety and social anxiety disorder

### Self-directed perception of gaze

In recent years, several studies have investigated the perception of self-directed gaze in order to quantify the perception of mutual gaze in social anxiety. Initial work used the previously described “cone of gaze” paradigm to investigate the self-directed perception of gaze cues in SAD (Gamer et al., 2011). In half of the trials an additional task-irrelevant looker was presented. The results provided support that patients with SAD exhibit an enlarged self-directed perception of gaze directions, but only in the presence of a second virtual looker. The magnitude of this effect was positively correlated with the severity of social anxiety symptoms.

Subsequent work investigated dimensional relations between social anxiety and the perception of gaze directions in a non-clinical sample, while also addressing the specific role of facial emotional expressions (Schulze et al., 2013). Severity of social anxiety was positively correlated with the self-directed perception of other individuals’ gaze, especially when the “lookers” exhibited a neutral or negative (i.e., angry, fearful) facial expression. In addition, response latencies negatively interacted with symptoms of social anxiety, presumably reflecting an increased avoidance of direct gaze. Similar findings were reported by Jun et al. (2013) who assessed self-directed gaze perception using male facial stimuli in students with high and low social anxiety. An increased cone of gaze was found only in male students with marked social anxiety, possibly because male students experienced greater discomfort when being looked at than females (see also Jun et al., 2013, for a discussion of possible interactions between the sex of “lookers” and “observers” in mutual gaze perception).

Notably, enhanced self-referential perception of gaze directions was also demonstrated in more ecologically valid experimental setups with alive target stimuli. Harbort et al. (2013) studied the effects of real persons and virtual heads on gaze perception. The findings underpinned that the CoDG was generally increased in SAD, but that effect sizes were larger in the *Real-Person-Condition* than in the *Virtual-Head-Condition*. The widening of the gaze cone in the *Real-Person-Condition* was suggested to be a consequence of higher arousal in SAD patients when confronted with a real person. In line with the proposed role of arousal, stress-induced increases in cortisol levels were previously shown to increase feelings of being looked at (Rimmele and Lobmaier, 2012). A face-to-face situation was also used by Honma (2013) who found the range of gaze directions perceived as self-directed to be much larger than the actual amount of eye contact and perception of mutual gaze was accompanied by greater pupil dilations (see also Honma et al., 2012). In this study, severity of social anxiety was positively correlated with perceived eye contact and pupil dilation.

Harbort et al. (2013) assessed the effects of Cognitive Behavioral Therapy (CBT) on gaze perception. Patients with SAD were tested prior to standardized CBT and again after approximately 24 therapy sessions had been completed. Prior to psychotherapeutic treatment, patients with SAD were characterized by increased perceptions of gaze as being self-directed. Intriguingly, after CBT patients with SAD did not differ from healthy controls, suggesting that interventions aiming at reducing SAD symptoms lead to a normalization of the gaze cone. These findings still need to be considered preliminary since the interaction of group and assessment time failed to reach significance; several alternative explanations might thus account for the observed pattern.

In sum, available studies in SAD demonstrated an abnormal perception of mutual gaze, providing a quantification of the intense feelings of being looked at. Findings unanimously** demonstrated an enhanced self-directed perception of gaze, particularly for negative and neutral facial expressions. Further studies are needed to investigate whether the cone of gaze changes due to psychotherapeutic interventions.

### Threat perception and avoidance of mutual gaze

Clinical observations suggest fear and avoidance of *direct* eye contact to be prominent characteristics of SAD. Yet, empirical evidence on threat-related perception and avoidance of direct gaze compared with averted gaze is still scarce.^1^

Initial studies provided some support that mutual gaze is feared and avoided in social interactions (e.g., Daly, 1978; Baker and Edelmann, 2002). These findings are however limited because subjective observations were used as dependent measures. Objective evidence for an avoidance of salient facial features was first provided by studies using eye-tracking to investigate visual responses to static images with direct gaze. Comparing visual scanpaths of emotional facial expressions in patients with SAD and healthy controls yielded an active avoidance of salient facial features such as the eye region in SAD. This was particularly reflected in reduced number and duration of fixations of the eye region while a “hyperscanning” strategy was exhibited for remaining facial features (Horley et al., 2003, 2004). This distinct visual scanning behavior was most prominent for expressions of threat, whereas group differences were least pronounced in response to neutral or happy facial expressions (Horley et al., 2003, 2004). Moukheiber et al. (2010) later replicated these results, finding less fixations and shorter dwell times on the eye region in SAD compared to healthy individuals. Again, group differences were most notable for expressions of social threat (i.e., anger and disgust). A reduced number and duration of fixations upon the eye region were also reported when SAD patients received social feedback (Weeks et al., 2013).

While these studies demonstrate an avoidance of the eye region, questions remained unanswered to what extent others’ gaze directions differentially affect avoidance behavior in SAD. This question was recently addressed by means of the Approach-Avoidance Task. In social anxiety, behavioral avoidance of angry faces was present only when coupled with direct gaze (Roelofs et al., 2010). Notably, administration of oxytocin facilitated approach behavior towards angry faces with direct gaze in socially anxious individuals (Radke et al., 2013). In a related line of research, fixation behavior was investigated in response to animated video clips of faces with direct or averted gaze (Wieser et al., 2009a). In high socially anxious participants longer fixations on the eye region were observed, although effects were only marginally significant. Additionally, heightened physiological arousal in socially anxious individuals was found for direct compared to averted gaze suggesting that mutual gaze is perceived as threatening. In line with this interpretation, increased startle reactivity was observed the very moment a virtual audience directed their eye gaze and attention towards individuals with SAD who had to deliver a speech (Cornwell et al., 2011). A virtual-reality environment was also used by Wieser et al. (2010) to scrutinize the interplay between gaze directions, interpersonal distance, and sex of the interaction partner on avoidance behavior. Socially anxious individuals were found to avoid eye contact and to show increased backward head movements in response to male avatars with direct gaze.

Further evidence for the threatening quality of direct gaze was obtained by functional neuroimaging studies in SAD (see Etkin and Wager, 2007 for a meta-analysis of neuroimaging and emotion processing in SAD). In a preliminary study comparing neural responses to direct and averted gaze, patients with SAD were found to exhibit greater activation in parts of the fear circuitry including the amygdala, insula, and anterior cingulate cortex (Schneier et al., 2009). Additional eye tracking results indicated that SAD patients show a greater avoidance of the eye region in stimuli with direct compared to averted gaze than healthy controls. In a subsequent study, neural responses to direct and averted gaze were assessed before and after intervention with paroxetine in patients with generalized SAD (gSAD; Schneier et al., 2011a). At baseline, gSAD patients showed greater activation than healthy controls in brain regions related to self-referential processing and emotion regulation such as cortical midline structures of the ventromedial prefrontal cortex and the posterior cingulate cortex, when looking at direct versus averted gaze. However, fixation of the eye region did not differ significantly between gSAD patients and healthy controls. Pharmacological treatment resulted in a normalization of brain activation in response to direct gaze.

In contrast to the studies reviewed above, recent electrophysiological evidence suggested a specific processing bias for averted gaze in social anxiety as implied by enhanced late positive potentials and (marginally significant) higher amplitudes of the P100 in response to averted gaze (Schmitz et al., 2012). These authors proposed that direct gaze might only be perceived as threatening when coupled with negative facial expressions, whereas neutral expressions with averted gaze might rather signal disinterest.

Taken together, there is ample evidence that mutual gaze is perceived as threatening by socially anxious individuals. However, findings are less consistent regarding the avoidance of mutual gaze in SAD. While several studies demonstrated an avoidance of the eye region when coupled with direct gaze, some studies failed to observe group differences and one study even reported prolonged fixation of the eye region. Future research suggestions will be discussed in the final section.

## Summary and Future Research

Clinical observations suggest abnormalities in gaze perception to be important for SAD. In accordance with such claims, findings from analog samples and clinical populations demonstrated a greater cone of gaze and a pronounced fear of direct eye contact in social anxiety. In addition, recent findings suggest that individuals with SAD avoid mutual gaze, but these results are less consistent.

In socially anxious individuals, a biased self-referential perception of gaze directions may underlie the fear of being the center of attention and cause uneasiness and discomfort. Specifically, biased perceptions of mutual gaze may lead socially anxious individuals to appraise a situation as social, which results in a heightened processing of the self as a social object, ultimately resulting in a negative cascade of somatic, cognitive, and behavioral consequences (Clark and Mcmanus, 2002). The avoidance of eye contact in social anxiety may be understood as an attempt to avoid signs of social threat and to regulate excessive fears of being evaluated. This avoidance behavior may contribute to the maintenance of SAD by negatively reinforcing expectations and fears of social encounters. Alternatively, taking into account findings of gaze aversion in social anxiety, it is also conceivable that SAD patients fail to extract relevant cues from the eye region. This factor may lead to abnormal perceptions of being looked at. A promising direction for future studies may therefore be to combine eye-tracking methods with paradigms of mutual gaze perception to further disentangle causes and consequences of abnormal gaze perception/behavior in social anxiety.

Measuring scan paths by means of eye-tracking is a highly ecologically valid method to assess overt gazing behavior. Hence, eye-tracking methods seem highly suitable to study avoidance of mutual gaze in individuals with social anxiety. In addition, such methods also allow studying approach-avoidance behavior in response to more ecologically valid stimuli, such as films or crowds of individuals (Lange et al., 2011). Ultimately, gaze measures may present objective benchmarks for the evaluation of psychotherapeutic treatment approaches for SAD. More specifically, scan paths may potentially be used as objective measures for avoidance behavior in social anxiety. Although the avoidance of mutual gaze is considered a behavioral marker of SAD (cf. Weeks et al., 2013), current findings are less consistent in this regard. A possible explanation for these inconsistencies may be that in most studies only *time-averaged* fixation behavior in response to direct gaze was analyzed. However, behavioral studies mainly suggest a hypervigilant-avoidant *time-course* of attention in social anxiety. In comparison to non-anxious individuals, threatening social information is detected earlier by socially anxious individuals (hypervigilance) and is followed by attentional avoidance of such stimuli (e.g., Wieser et al., 2009b). More fine-grained analyses and paradigms might thus help to disentangle differential effects of early and late processes on fixation behavior in SAD (see also Bar-Haim et al., 2007; Armstrong and Olatunji, 2012).

Further research is needed to assess the diagnostic value of abnormalities in gaze perception as possible behavioral indicators of SAD. To date, statements regarding the diagnostic potential of such measures are substantially limited since none of the studies included a clinical comparison group, comprising for instance individuals with symptoms of autism, or schizophrenia who also exhibit abnormal gaze perception (Kliemann et al., 2010; Clark et al., 2013). It remains therefore unclear whether avoidance and fear of gaze are specific for socially anxious individuals or whether they are general signs of psychopathology and interpersonal dysfunction. Furthermore, the specific functions of gaze avoidance and its effects on states of social anxiety remain to be clarified. Langer and Rodebaugh (2013) recently demonstrated avoidance of eye-to-eye contact to be an ineffective strategy for the regulation of anxiety in social phobic individuals.

In sum, recent findings highlighted abnormal gaze perceptions in social anxiety. In particular, socially anxious individuals were characterized by a greater self-referential perception of gaze direction along with a pronounced fear of direct eye contact.

## Conflict of interest statement

The authors declare that the research was conducted in the absence of any commercial or financial relationships that could be construed as a potential conflict of interest.

## Footnotes

^1^Note that the present review is focused specifically on *gaze perception*. For studies using eye-tracking in SAD to investigate attention mechanisms in general, see Armstrong and Olatunji (2012).
